# Preliminary Assessment of the Quality of Life and Daily Burden of Caregivers of Persons with Special Needs: A Questionnaire-Based, Cross-Sectional Survey

**DOI:** 10.3390/ijerph20032012

**Published:** 2023-01-21

**Authors:** Andréa Lanzillotti Cardoso, Geraldo Oliveira Silva-Junior, Luciana Freitas Bastos, Ana Luiza Medeiros Cesar, Leila Goes Serrano, Arkadiusz Dziedzic, Bruna Lavinas Sayed Picciani

**Affiliations:** 1 Department of Preventive and Community Dentistry, School of Dentistry, Rio de Janeiro State University, Rio de Janeiro 20551-031, RJ, Brazil; 2 Graduate Program in Dentistry, Health Institute of Nova Friburgo, Fluminense Federal University, Nova Friburgo 28625-650, RJ, Brazil; 3 Department of Diagnosis and Therapeutics, School of Dentistry, Rio de Janeiro State University, Rio de Janeiro 20551-031, RJ, Brazil; 4 Department of Conservative Dentistry with Endodontics, Medical University of Silesia, 40-055 Katowice, Poland

**Keywords:** caregivers, quality of life, disabilities, special needs, autistic spectrum disorder, survey

## Abstract

Caregivers of persons with special needs (PSN) experience a variety of burdens and elevated levels of stress and anxiety throughout their lives, leading to a physical, psychological, emotional, social, and financial overload. This analytical study with a cross-sectional design and a quantitative approach aimed to appraise quality of life (QoL), reflecting the daily workload of informal family caregivers of PSN. Methods: Four structured, validated questionnaires were utilised: sociodemographic, WHOQOL-bref, Zarit Burden Interview, and Functional Independence Measure Scale in 60 anonymous volunteered respondents. Results: The informal caregivers were middle-aged mothers (81.7%), married (55%), stay-at-home spouses (60%) with high school degrees (51.6%), providing a care for their relatives with special needs for more than 20 years (41.8%). Most of the PSN were diagnosed with autistic spectrum disorder (ASD, 61.8%), had a wide spectrum of intellectual deficits, and required constant support for their basic needs. They were mainly adolescent males without physical limitations (83.4%) on disorder-specific medications (90%). The study revealed that those caregivers had a median perception of QoL considering four essential domains, with a highest score recorded for the physical domain (64.3 +/− 16.1 SD). A moderate burden level prevailed, revealing neither a correlation between the workload expressed by caregivers and the patient’s functional capacity, nor in the performance of daily self-care tasks (Spearman correlation test *p* > 0.05), apart from the environmental domain (mild correlation = 0.335, *p* < 0.05). Conclusions: The reported average level of overload associated with QoL of informal caregivers exists, affecting a vast proportion of the respondents. The absence of a direct association between workload and the functional capacity/daily self-care tasks can be related to the significant personal dedication of family caregivers, regardless of their socioeconomic status.

## 1. Introduction

Relatives acting as informal caregivers of people with special needs tend to experience a variety of burdens, elevated levels of stress, and long-term concerns over the course of their lives [1,2] associated with permanent physical exhaustion, social isolation, financial burden, and psychological strains, as well as common constraints due to insufficient support. Although caregiving is deemed a parental duty/responsibility when dealing with PSN, parents may be at a greater risk of mental health disorders, including depression and anxiety. Reportedly, caregivers have multiple concerns, spanning financial expenses to the wellbeing and social acceptance of their dependents. Despite constituting crucial support for PSN, informal caregivers may not have the adequate knowledge to maintain satisfactory care for relatives with special needs. Other components substantially compound caregivers’ QoL, such as PSN’s impaired motor coordination, behavioural issues, and reluctance to collaborate, as well as limited access to dedicated special care. With regard to oral health, this aspect can be severely compromised, since routine oral hygiene and self-care in vulnerable groups of PSN are not considered priorities on account of other daily challenges. The provision of specialist dental care for PSN, including treatment under sedation or general anaesthesia, is deemed inadequate worldwide. PSN require a substantial support to maintain stable oral health in the form of toothbrushing carried out regularly by carers/caregivers. The additional prophylactic aids, such as interdental cleaning and the use of fluoridated topical measures, help to achieve this goal. The use of xerostomia-induced medications that alter salivary production may have a negative impact on oral health, causing an increased risk of dental caries and periodontal diseases compared to the general population [3,4,5,6]. Oral health-related problems in PSN resonate strongly with long-standing burdens in caregivers’ lives.

The high prevalence of oral diseases as a result of low compliance with the daily oral hygiene routine obliges health professionals to provide intensified oral hygiene instructions for PSN [7]. Reportedly, families of PSN face major obstacles associated with oral hygiene regimes in PSN, due to the lack of sufficient advice regarding domestic oral hygiene [8]. Undoubtedly, the family plays an essential role as the ‘core foundation’ for maintaining appropriate domestic oral hygiene in PSN and arranging adequate dental care for their relatives with special needs. The constant involvement in care, provision of specialist medical care, and the challenges associated with health-related problems are deemed persistent burdens and daily overload that negatively affect caregivers [1,9,10]. A persistent overload related to multifaceted care directly influences the quality of life of caregivers, reflecting the non-verbalised expectations and paradigms they have about their own life goals and values [8,9,10]. Family caregivers are of special interest to social and public health science, given the impact that the act of personal caring has on their health and living conditions, as well as the possibility that they evolve into ‘hidden patients’. Caregiving can be emotionally and physically demanding considering long-term physical and mental health, while on the other hand, it is deemed rewarding. It is essential to recognise when it is becoming overwhelming and take steps to prioritise one’s own wellbeing. Thus, it is justified to investigate, in depth, the QoL and work overload of informal caregivers who face a life-long burden.

Due to the reported deficit in the evidence-based knowledge regarding the various aspects of QoL reflecting the existing burden of informal caregivers, it seems prudent to pursue robust research projects resulting in the delivery of data supporting dedicated care in underserved persons with special requirements [9,10]. Hence, this implies a rationale to support research that provides evidence for future public health policies in diverse populations [1,4], collaborating on the enhancement of services equally dedicated to patients and caregivers. They can favour targeted health programmes and social inclusion involving PSN.

This preliminary survey-based study with an analytical approach aimed to assess and associate QoL with the workload of family caregivers of PSN, specifically those with moderate and severe learning disabilities and mental health disorders.

## 2. Materials and Methods

### 2.1. Study Design, Participant Allocation, Inclusion and Exclusion Criteria

As exploratory research of a descriptive nature, this multifaceted study adopted a cross-sectional design along with a comprehensive quantitative approach, utilising four validated questionnaires. Data collection took place from July to December 2021, with 170 new patients being treated and 80 patients able to complete the form. Of these, only 60 informal caregivers of patients diagnosed with a mental disorder agreed to participate in the research. In this way, the research took place with a non-random sample consisting of 60 informal caregivers of people diagnosed with disabilities and with various special needs, namely, with moderate and severe learning disabilities, without age restrictions. The study was conducted at the Dental Center for Radiology and Care for Patients with Special Needs at Policlinic Piquet Carneiro, Rio de Janeiro State University, Rio de Janeiro, Brazil. The informal caregivers, defined as the person taking on the total or greater responsibility for the care provided to the PSN without financial reward/earnings, was allocated as the primary respondent for this study.

The inclusion criteria defined that the respondents were the informal caregiver of a person with a diagnosed learning disability, able to understand and answer the questionnaire’s questions, and were not receiving any type of remuneration for performing the care task (unpaid care). The selection of a ‘learning disability’ diagnosis was based on the most frequently recorded medical diagnosis in persons who required specialist dental treatment under sedation or general anaesthesia due to non-compliance and/or inadequate cooperation. The incompletion of the questionnaire’s answers and any financial involvement associated with the care provided were the main exclusion criteria.

### 2.2. Ethical Considerations

The project was approved by the Research Ethics Committee of Rio de Janeiro State University under CAAE: 42335320.7.1001.5259 and authorised under number 4.628.928. The study design and protocol were fully compliant with the Strengthening the Reporting of Observational Studies in Epidemiology (STROBE) guidance. The anonymous identity of the caregivers and PSN was implemented to control Hawthorne’s effect and survey bias. Informed, valid, written consent was obtained from all subjects involved anonymously in this survey.

### 2.3. Questionnaire Instrument Characteristics

All participants completed a sociodemographic questionnaire, the WHOQOL-bref, the caregiving burden scale called the Zarit Burden Interview (ZBI), and the Functional Independence Measure (FIM). The sociodemographic questionnaire was used to collect sample characterisation data, including (i) the caregiver’s sex, age, age group, education, marital status, family income, relationship with the patient, occupation, residence, and time of care, and (ii) the patient’s sex, age, age group, diagnosis of the main disease, use of medication, and physical limitations.

The caregiver’s quality of life was stratified through the World Health Organization’s Quality of Life Questionnaire (WHOQOL-bref in the abbreviated version) consisting of 26 closed questions encompassing four essential domains: (i) physical, (ii) psychological, (iii) social relations, and (iv) environment [11,12]. The WHOQOL-bref has five-point Likert scale responses: “very bad to very good” (assessment scale), “very dissatisfied to very satisfied” (assessment scale), “nothing to extremely” (intensity scale), “nothing to completely” (capacity scale), and “never to always” (frequency scale) [12,13]. Each domain comprises questions with response scores ranging from one to five. A higher score indicated a better the perception of QoL. The results were presented as four independent domains, and the domain score was calculated using a raw scale. The values were converted and varied between 0 and 100 (0 is the worst and 100 is the best value for each dimension).

To assess the burden of the main caregiver, the Zarit Burden Interview (ZBI) scale was used [14] containing 22 items that include information about health, financial status, emotional status, social life, and personal life. Similar to the WHOQOL-bref, the answers were given on a five-point Likert scale, from 0 to 4, where 0 = never, 1 = almost never, 2 = sometimes, 3 = often, and 4 = almost always. This score revealed the frequency of each item, with a higher score corresponding to a greater burden, according to the following cut-off points: less than 21 corresponds to the absence of a burden; between 21 and 40 indicates a moderate burden; between 41 and 60 indicates a moderate to severe burden; and greater than 61 corresponds to a severe burden [15,16].

To conduct the assessment of the functional capacity of PSN and the performance of daily self-care tasks, the Functional Independence Measure (FIM) [17] was used. The FIM scale is organised by classifying a patient’s ability to perform an activity versus a patient’s need for assistance from another person or resource of adequacy in a set of 18 tasks, referring to the subscales of self-care, sphincter control, transfers, locomotion, communication, and social cognition [18]. The FIM is divided into two dimensions (motor and cognitive), which differentiate the functional independence of patients, measure the degree of request for patient care, and reflect the main needs in terms of help or assistance. Each of the activities was evaluated and received a score from 1 to 7, being categorised as follows: (1) total assistance (the individual receives 100% assistance); (2) maximum assistance (the individual can perform up to 25% of the task); (3) moderate assistance (the individual can perform up to 50% of the task); (4) minimal assistance (the individual can perform up to 75% of the task); (5) supervision (the individual performs the task under supervision); (6) modified independence (use of auxiliary resources to perform the task); and (7) complete independence (performs the task safely and in a satisfactory time). The total recorded score ranged from 18 to 126, with the lowest score corresponding to greater dependence, according to the following cut-off points: less than 18 corresponds to complete dependence; between 19 and 60 indicates maximum to moderate dependence; between 61 and 103 indicates minimal dependence on supervision, stimulation, or preparation; and between 104 and 126 corresponds to modified independence.

### 2.4. Statistical Analyses

Descriptive and inferential statistics were used for the data analysis, applying Spearman’s correlation test to deal with the ordinal data [19]. The coefficient ranges from −1 to +1, where 1 represents a perfect monotone relationship between the two variables. Accordingly, +1 represents a perfect directly proportional correlation, while −1 represents a perfect inversely proportional correlation. In addition, the following parameters were observed: 0–0.1 = insignificant correlation; 0.1–0.39 = weak correlation; 0.4–0.69 = moderate correlation; 0.7–0.89 = strong correlation; and 0.9–1 = very strong correlation [19]. The level of statistical significance was set as *p* < 0.05.

## 3. Results

### 3.1. General Characteristics of Study Group and PSN

This study included sixty participants who represented the group of informal caregivers as pre-defined in the cross-sectional survey inclusion criteria. Regarding gender, females were the most prevalent, making up 81.7% of the participants. Their age ranged from 18 to 80 years, with a mean of 41 years (SD = 13). Most respondents (51.6%) had a high school education and 55% of them were married (Table 1). Family income was distributed more frequently in the range of two to five minimum wages in 31 (51.7%) respondents, with the mother being the main family caregiver (81.7%) and declaring ‘home keeper’ as their main occupation in 61.6% of cases. In addition, 60% had their own home and 31.7% had been informal caregivers for more than 20 years (Table 1). All PSN had some level of intellectual deficit requiring support for their daily activities and basic needs. The most common clinical diagnoses were autistic spectrum disorder (ASD) (60%), schizophrenia (13.3%), and trisomy 21 (11.7%), with the majority being males (65%) aged between 11 and 20 years old (28.3%), without physical limitations (83.4%). Expectedly, 90% of the PSN were subject to pharmacotherapy for chronic conditions (Table 2).

### 3.2. Quantitative Evaluation of QoL, Workload, and Burden

To assess the caregivers’ QoL, the WHOQOL-bref was used, with the general average of the three domains having scores close to 50, demonstrating a median perception of QoL, with the physical domain (mean 64.3, 16.1 SD) having the highest score and reflecting a substantial impact, followed by the psychological domain (55.4, 11.3 SD, Table 3). Concerning the caregivers’ workload, according to the ZBI scale, the scores ranged from 9 to 60 points, with a mean of 35 points (SD = 9.5), being a moderate burden (Table 3). Analysing the ZBI questionnaire-derived data as a categorical scale, a moderate burden prevailed with 68.3%, followed by a moderate to severe burden (21.7%), no burden (8.3%), and a severe burden (1.7%), respectively. Less than 10% of caregivers declared the absence of burden (< 21 score). In relation to the assessment of the functional capacity of PSN and the performance of daily self-care tasks, the FIM scale ranged from 25 to 125 points, with a mean of 93.1 (SD = 23.3). The results showed maximum dependence on supervision in the cognitive area and minimum dependence on the motor components as declared by the caregivers (Table 3).

The Spearman test, applied to correlate the workload indicated by caregivers with the PSN functional capacity and the performance of daily self-care tasks, showed no significant correlation between the results derived from the two scales. A moderate inverse correlation between the burden (Zarit) and the assessed domains was found, represented by the WHOQOL-bref. The correlation between the FIM and the WHOQOL-bref domains was not significant, except for the environment domain, which showed a weak correlation (0.335, Spearman correlation, *p* < 0.05, Table 4). Conversely, there was a moderate statistically significant correlation between the physical and psychological domain, and the physical and interpersonal domain; a strong relationship between the physical and environment domain; a moderate relationship between the psychological and interpersonal domains, and the psychological and environment domain; and a moderate relationship between the interpersonal and environment domain (Table 4, *p* < 0.05).

## 4. Discussion

This non-intervention analytical study preliminarily evaluated the complex interactions and effects of individual care delivered by informal caregivers to PSN, with a specific aim of assessing multiple aspects of caregivers’ quality of life. The presented study is the first to investigate several variables related to the characteristics of the persons with special needs and the caregivers’ individual perception of existing burdens, in the broad context of quality of life.

While many forms of the aspects of direct, continuous care dedicated toward patients with special requirements exist, the care provided to PSN is associated with a lifetime of dedication, which raises the question of whether this can lead to an overload of work and whether it influences the personal perception of one’s QoL. In our study, middle-aged (41–50) females, specifically mothers, were the main caregivers considering the fact that they were responsible for the permanent support and assistance of care-receivers, as similarly noted in several other studies [9,20,21,22,23]. Predominantly, the cultural factor tends to impose the role of informal caregiver onto women [9]. It has been suggested that, as women have “historically” been the foundation of psychological support of the family, it is natural that they take on the role of informal caregiver.

A recent study showed that mothers of persons diagnosed with autistic spectrum disorder represent the main caregivers, who report a relatively high burden particularly associated with a care towards children aged 6 and 12 years, increasing in correlation with the severity of autism. Considering reports of married women taking care of their child with special needs, the results of this study coincide with those found by other authors [21,24], who revealed that the wellbeing of married female caregivers may be maintained, as their ability to share tasks with their husbands can also positively affect their perception of QoL [23]. These interesting findings were also observed in our study, despite the fact that the family income was in the range of two to five minimum wages, allocating families to a group of low socioeconomic status and compromising their livelihood. Undoubtedly, the act of care provision may prevent caregivers from looking for full-time work.

Moreover, the main occupation reported was “home keeper” since, in dedicating time to the PSN, the caregivers were only able to take on informal occupations and job tasks, which could be due to 48% of caregivers being uneducated or educated below high school level. Here, our study disclosed that the informal caregivers represented by the sample had been looking after a PSN for a long time and had taken on the assignment of care for more than 20 years. It is expected that these factors contributed positively to the ‘overestimated’ perception of QoL, as maturity brings a different outlook on life’s challenges, and the security of property ownership decreased the level of uncertainty among the caregivers. However, the potentially occurring Hawthorne’s effect could also prompt overestimation. Reference can be made to the study by Giovannetti et al. [25], who also observed that Italian long-standing caregivers of patients in a vegetative state or minimally conscious state reported a significantly lower level of anxiety compared to persons who became caregivers shortly after an acute medical event.

Concerning the PSN, diverse intellectual disabilities also require an individually applied level of assistance for daily activities. Of the individuals observed, 60% were diagnosed with autistic spectrum disorder, having an average age of 21 years, without major debilitating physical limitations. In regard to ASD, despite the absence of physical limitations positively impacting the QoL of the caregivers [23], they nonetheless indicated insecurity and anguish resurfacing over the years [25], as the various sensory disorders and comorbidities exacerbate the difficulty of social interaction and restrict individual autonomy.

The analysis of the caregivers’ QoL showed an average of the domains with scores close to 50, with the physical domain having the highest value, revealing a lower impact in this area. The physical domain refers to issues related to pain, physical discomfort, and fatigue, energy, and satisfaction with daily activities and work, as well as medication dependence. Interestingly, most caregivers claimed not to have any health problems and considered their health status to be good/stable. However, this differs from the study presented by Pandey and Sharma [26], which concluded that providing 24/7 care to a PSN for many years considerably affects the caregiver’s physical health. Patel et al. [9] added that a general health status can be impaired mainly in low-income populations. The psychological domain, which reached a score of 54.5%, related to positive thinking about caregivers’ affective and cognitive condition and was lower than the results obtained in previous studies [9,23]. Our results seem contrary to the research carried out by Scherer et al. [27], who indicated high levels of depressive symptoms among parents of children with disabilities. On the other hand, evaluating the social relationships domain, which presented a score of 53.3%, revealed that the little or no family support and sparse social relationships of these families negatively influence this domain [9,22]. As with other studies [9,23], the environment domain reflected the lowest score of 48.8%, addressing unresolved issues related to security, housing, financial resources, health services, leisure, and transport. This domain would require the presence of public policies that guarantee the proper functioning of these services [21].

In analysing the results based on the ZBI scale, the fact that more than 50% of the respondents scored above 35 points may indicate an increased risk of mental health disorders. Schreiner et al. [28] observed that a ZBI cut-off score ranging from 24 to 26 has significant predictive validity for identifying caregivers susceptible to depression. The authors stated that these caregivers could be reliably identified by administering the ZBI scale alone and their data confirm the anxiety-related overload shown in other studies [21,23]. The moderate overload found seems consistent with the findings of several other studies [26,27,28], although Moreira et al. [29] and Estrada-Hernandez [23] demonstrated more intense overload. Despite a moderate burden being identified in our study, the caregivers did not declare substantially elevated anxiety; on the contrary, they showed general compliance and satisfaction with access to dental care services dedicated to PSN. Predictably, over the years, caregivers learn to adapt to their duties and realities, and what once seemed challenging becomes eventually a norm. Undoubtedly, it has been demonstrated that variables such as child behavioural problems, difficulties of inclusion in school, deficits in social interactions, and adverse economic conditions can contribute to the caregiver’s burden [9,21,22,23,30]. Despite this, it has been observed that informal caregivers add the act of caring to their daily activities and may even express positive emotions over the “tasks performed” in reflecting that the child is well taken care of [31]. What is more, it seems that caregivers with a higher level of education are more self-confident in dealing with various challenging situations affecting their life [17]. The correlation between the QoL instrument and the work overload instrument points to a moderate inversely proportional relationship, as in the findings of several other authors [17,21,23,27,31]. Despite the obvious burden reflected by the Zarit scale, many caregivers can also waive any association of this nature when it applies to their loved ones. This fact may be related to the high score of the motor component of the FIM (70.1) because of the lack of dependability of caregivers.

Interestingly, our study was unable to disclose a correlation between the caregivers’ workload and the patient’s functional capacity and the performance of self-care tasks, corroborating Naing’s results [16]. It must be noted, however, that such results differ from Ten Hoopen et al. [10], who found a significant association between the degree of dependence and the caregiver’s workload. Notably, according to the caregivers, 83% of PSN did not have any considerable physical limitations and, as a result, the motor component of the FIM did not influence the caregiver’s primary perception of overload. The correlation between the FIM score and most of the WHOQOL-bref domains was also not significant, except for the environment domain, which showed a weak correlation (0.335, Spearman correlation, *p* < 0.05). These findings indicate that the functional capacity of the individuals cared for does not interfere with the perception of the QoL of their caregivers, who do not experience the major fatigue associated with the physical demands in their daily lives, even if the cognitive area has been affected.

### 4.1. Limitations and Strengths

Firstly, the use of a convenience sample from the same service (a single-service study) and the small sample size are relative limitations of this study. This might affect the allocation of respondents and distribution of essential variables, with a direct impact on the obtained results. The relatively uniform group of persons with special needs without major physical disabilities and impairments may not be directly comparable to other populations. The results from single selected populations cannot be directly extrapolated taking into consideration a global scale. In addition, it could be assumed that caregivers unintentionally tended to underestimate their daily workload and subsequently overestimate their QoL while completing this survey. The utilisation of validated and standardised quantitative tools (questionnaires) and a homogeneous study group are the main strengths, supporting the data reliability and reproducibility.

### 4.2. Implications

The results obtained should prompt further well-designed, multi-centre, and robust studies assessing caregivers’ QoL and providing comprehensive data regarding their wellbeing worldwide in the context of currently existing social and public health strategies. A large sample multivariate analysis conducted in the future may deliver additional input concerning multi-directional associations between various crucial components of QoL. Such evidence-based data are expected to help commissioners and policymakers implement a widespread public health intervention, including the improvement of primary and secondary care for persons with various spectra of special needs who require permanent support. Adequate services providing support and counselling, as well as the sufficient and effective management of caregivers’ burdens, should be widely implemented as an integral element of routine care dedicated to persons with disabilities.

## 5. Conclusions

With the limitations of the questionnaire-based survey, this study demonstrated that married and middle-aged mothers as the main caregivers of persons with special needs had a median perception of quality of life, declaring the physical domain as the highest value. The correlation between their quality of life and care-related overload suggested a moderate inversely proportional relationship, with no obvious correlation between care overload and the patient’s functional capacity or the performance of daily self-care tasks. In addition, the results showed maximal cognitive dependence and minimal motor dependence considering supervision, stimulation, or preparation in most of the PSN. Arguably, some caregivers reject any association of a burden with the care they provide to their loved ones, instead facing their daily challenges with resilience. It is important for caregivers to communicate their needs and boundaries, seeking professional help from support groups when necessary.

## Figures and Tables

**Table 1:** Distribution of the study group according to the sociodemographic profile of caregivers.

Variable	Category	Total	
		*N* = 60	%
Sex	Male	11	18.3
	Female	49	81.7
Age group	18 to 30 years old	5	8.4
	31 to 40 years old	12	20
	41 to 50 years old	23	38.3
	51 to 60 years old	8	13.3
	>60 years old	12	20
Marital Status	Married/Stable Union	33	55
	Single	15	25
	Widow	9	15
	Divorced	3	5
Relationship with the patient	Mother	49	81.7
	Father	5	8.4
	Sibling	2	3.3
	Grandmother/Grandfather	2	3.3
	Uncle/Aunt	2	3.3
Education	No education *	12	20
	Primary education	12	20
	High School	31	51.6
	Graduate	5	8.4
Occupation	Home keeper	37	61.6
	Informal job	15	25
	Formal job	8	13.4
Family Income	Up to 1 minimum wage	29	48.4
	>1 up to 5 minimum wage	31	51.6
Residence	Own house	36	60
	Rent	12	20
	Relative’s house	12	20
Overall duration care	Up to 10 years	16	26.6
	>10 to 20 years	19	31.6
	>20 years	25	41.8

* Caregivers who also did not complete elementary school were included in this group.

**Table 2:** Sample distribution according to the sociodemographic and clinical profile of the persons with special needs.

Variable	Category	Total	
		*N* = 60	%
Sex	Male	39	65
	Female	21	35
Age group	0 to 10 years old	16	26.7
	>10 to 20 years old	17	28.3
	>20 to 30 years old	10	16.7
	>30 to 40 years old	14	23.3
	>40 to 50 years old	2	3.3
	>50 years old	1	1.7
Diagnosis	Autism Spectrum Disorder	36	60
	Schizophrenia	8	13.3
	Trisomy 21	7	11.7
	Isolated Intellectual Disability	5	8.4
	Hydrocephalus	2	3.3
	Cerebral Palsy	2	3.3
Medication	Yes	54	90
	No	6	10
Physical limitation *	Yes	10	16.6
	No	50	83.4

* When the individual needs help to perform a physical task or when the task is not performed.

**Table 3:** Sample distribution based on assessment of caregivers’ WHOQOL-bref domains, ZBI scale and FIM result.

Scales	Mean	Standard Deviation	Median	Minimum	Maximum
Domain Physical	64.3	16.1	66.1	28.5	96.4
Domain Psychological	55.4	11.3	58.3	25	79.1
Domain Personal Relationship	53.3	15.5	54.1	17.1	92.5
Domain Environment	49.8	12.2	47.1	22.1	75
Zarit Scale	35	9.5	35	9	60
FIM Scale	-	-	-	-	-
Cognitive	23.1	6.7	25.1	7	35
Motor	70.1	19.7	80	18	91
Total	93.1	23.3	100.5	25	

**Table 4:** Spearman correlation coefficients between WHOQOL-bref domains, Zarit scale and FIM result.

Questionnaire	Domain Physical	Domain Psychological	Domain Personal Relationship	Domain Environment	Zarit Scale	FIM Scale	FIM Cognitive	FIM Motor
Physical Domain		0.640 **	0.520 **	0.706 **	−0.507 **	0.202	0.015	0.142
Psychological Domain	0.640 **	1.00	0.588 **	0.619 **	−0.425 **	0.235	0.109	0.171
Personal Relat Domain	0.520 **	0.588 **	1.00	0.574 **	−0.655 **	0.236	0.164	0.188
Environment Domain	0.706 **	0.619 **	0.574 **	1.00	−0.551 **	0.335 **	0.222	0.238
Zarit Scale	−0.507 **	−0.425 **	−0.655 **	−0.551 **	1.00	−0.211	−0.184	−0.169
FIM Scale	0.202	0.235	0.236	0.335 **	−0.211	1.00	0.643 **	0.880 **
FIM Scale Cognitive	0.015	0.109	0.164	0.222	−0.184	0.643 **	1.00	0.348 **
FIM Scale Motor	0.142	0.171	0.188	0.238	−0.169	0.880 **	0.348 **	1.00

** *p* < 0.05.

## Data Availability

Not applicable.
